# Sand bed river dynamics controlling microplastic flux

**DOI:** 10.1038/s41598-024-80892-3

**Published:** 2024-11-27

**Authors:** Hazel Beaumont, Annie Ockelford, Phill Morris-Simpson

**Affiliations:** 1 School of Engineering, University of West of England, Bristol, UK; 2 School of Civil and Environmental Engineering, University of Liverpool, Liverpool, UK; 3 School of Applied Sciences, University of Brighton, Brighton, UK

**Keywords:** Microplastic flux, Bedform dynamics, Rivers, Sand sediments, Hydrology, Environmental impact

## Abstract

Microplastic contamination of river sediments has been found to be pervasive at the global scale and responsive to plastic and sediment bed properties, the flow regime and the river morphology. The physical controls governing the storage, remobilization and pathways of transfer in sand bed rivers remain unquantified. This means it is not currently possible to determine the risks posed by microplastic contamination within these globally significant river systems. Using controlled flume experiments we show that sand bed rivers can store up to 40% of their microplastic load within the sediment bed indicating that these environments can act as resilient sinks of microplastics. By linking bedform dynamics with microplastic transport characteristics we show that similarities exist between granular transport phenomena and the behavior, and hence predictability, of microplastic flux. Specifically, we demonstrate the inverse relationship between bedform celerity and microplastic retention within the bed can be used to predict microplastic flux. Further, we show that, in these environments, microplastic shape is more important than previously thought in controlling the fate of microplastics. Together, these findings are significant since they have important implications for the prediction and hence management of microplastic contamination in sand bed environments.

## Introduction

Rivers are the primary terrestrial conduits of plastic, delivering an estimated 0.8–2.7 million metric tonnes of plastics to coastal and marine environments each year^1^. However, river sediments also form important plastic reservoirs where microplastic (plastic particles less than 5 mm) concentrations have been found to be up to 600,000 times higher than those found in the overlying water column^2,3^. Whether microplastics are transported from or incorporated into a sediment bed is dependent on their properties, sediment bed properties, the flow regime and the river morphology.

Criteria for defining the onset of particle motion in alluvial rivers have largely been based on the work of Shields^4^ that estimated sediment flux in response to changes in average shear stress and sediment size. The Shields curve was originally developed to describe uniform sediments and as such, when applied to a range of grain sizes is characterised by considerable scatter linked to both grain and flow parameters^5^. Although governed by the same principles, predictions of microplastic transport in alluvial, fluvial environments, is also evidenced by considerable scatter on adapted versions of the Shields diagram^6,7^. This work showed that, in comparison with the Shields diagram, half of the microplastic particles moved earlier than natural sediments would, and therefore a higher microplastic transport rate can be assumed than would be determined with the theory from sediment transport. Differences here between alluvial sediments and microplastics were attributed to differences in density, shape, and size. Further, this work showed that the relative size effects between the sediment bed and microplastics was related to hiding-exposure effects.

Whether the riverbed acts as a source or sink of microplastic is controlled by flow regime and the properties of the sediment bed^8^. River discharge, especially peak discharge, plays a key role in moderating plastic flux in fluvial systems^9,10^. However, microplastic flux has also been shown to be temporally responsive to the flow regime where there are thresholds at which the sediment bed transitions from being a sink to a source of microplastics and back again^11^. In gravel bed rivers, these thresholds occur in response to changes in the relationship between the granular bed mechanics and the discharge characteristics, with microplastic flux controlled by active layer dynamics^12^. Specifically, under high flow, rivers act as sources of microplastic as plastic is mined from within the sediment bed and transported downstream. Conversely, under low flow conditions riverbeds act as resilient sinks for plastics where there is a tendency towards vertical exchange meaning a ‘loss’ from the exchange layer indicating a potential for their incorporation into long term sedimentary deposits. Given active layer dynamics vary depending on grain size and distribution there are likely to be significant differences between the processes controlling microplastic flux in sand bed rivers as compared to gravel bed rivers^13^.

Some of the biggest rivers in the world discharging sediment to the ocean are composed of sand grained material^14^. In these environments the sediment bed surface is characterised by bedforms with their dynamics being well described by bedform phase diagrams including a wide range of formulas to predict equilibrium bedform dimensions as a function of parameters such as flow strength, flow depth and sediment size^15–19^. Bedforms can be well preserved in the rock record and yield information about their formative processes^20,21^ however, the incorporation of plastics into the sand bed has been shown to influence bedform morphology and sediment transport processes^22^. Thus, we may need to adapt these relationships to account for plastic contamination such as to be able to better predict their likely propensity for incorporation into sedimentary deposits.

In this paper, we report a series of novel, mobile-bed laboratory flume experiments designed to explicitly quantify the relationship between bedform dynamics and microplastic flux in sand bed environments. In doing so, we highlight that sand bed rivers can store up to 40% of their microplastic load within the sediment bed. We show that, in these environments, microplastic shape is an important controlling factor in their mobility and is likely more important than relative size effects. We also show active layer dynamics control whether sand bed rivers act as a source or a sink of microplastics such that flux can be predicted by using the inverse relationship between bedform celerity and degree of microplastic retention within the bed. These findings have important implications for the prediction of microplastic contamination in sand bed environments.

## Results

### Microplastic dynamics

**Fig. 1:**
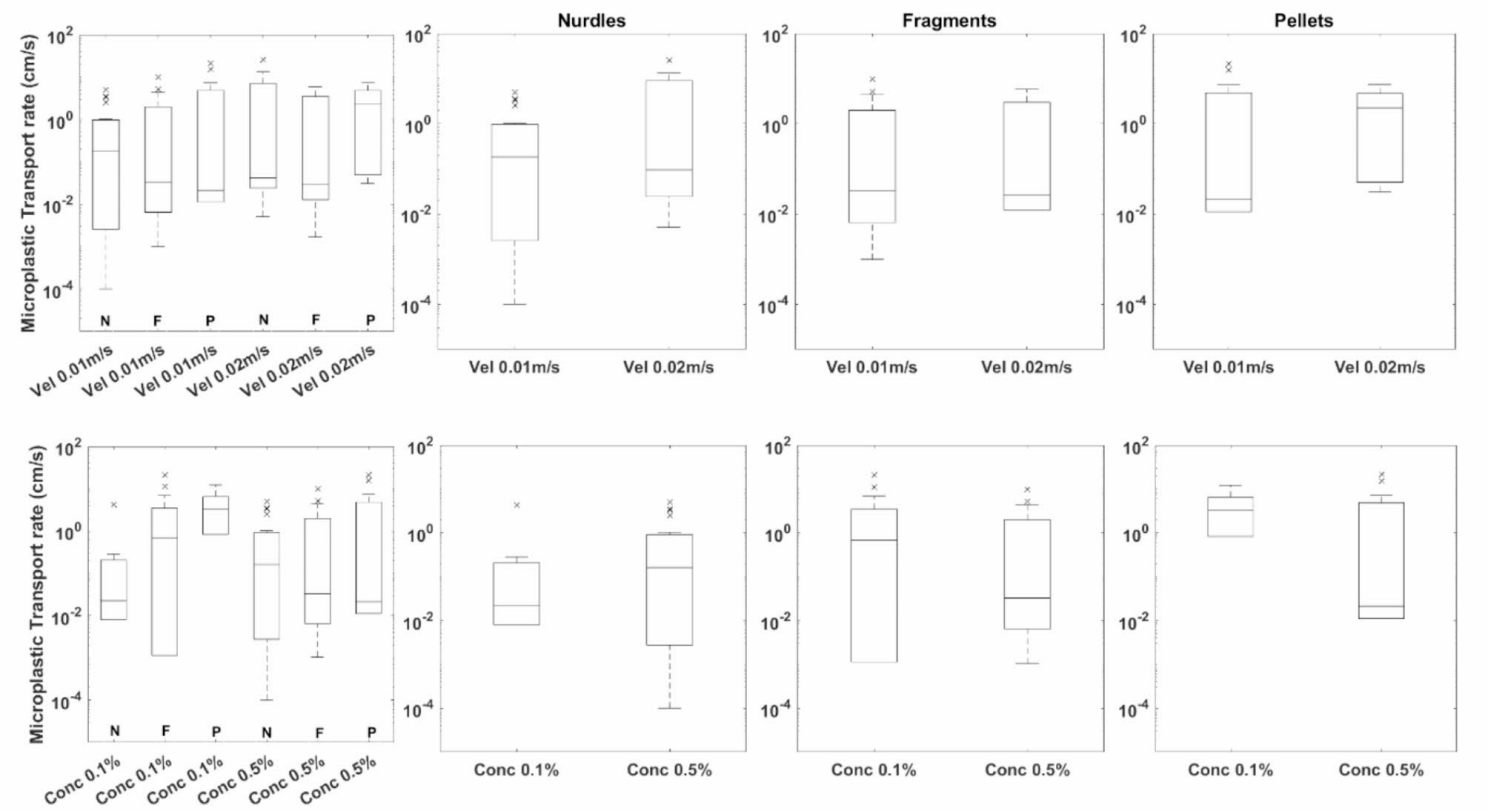
Distribution of microplastic transport rates for the three types of microplastic used within the experiments as a function of flow velocity and bulk microplastic concentration. The left-hand sub plots show all data for comparison with the three sub plots on the right grouped individually for each plastic type for velocity (top) and concentration (bottom). On all graphs the central mark indicates the median, and the bottom and top edges of the box indicate the 25th and 75th percentiles, respectively. The whiskers extend to the most extreme data points not considered outliers, and the outliers are plotted individually using the '*x*' marker symbol.

To understand the likely propensity for microplastics to be retained within a sediment bed it is first necessary to understand the dynamics of the plastic particles themselves, defined in these experiments by their transport rate and hop length. There are no consistent or statistically significant trends observed with microplastic transport rates in response to either changing flow velocity or bulk microplastic concentration (Fig. 1). Specifically, a 53% and 4% decrease in median transport rate was observed for nurdles and fragments as compared to a 116% increase for pellets as flow velocity increased. As bulk microplastic concentration increased, median microplastic transport rate increased 60% for nurdles but decreased by 69% and 86% for fragments and pellets respectively.

**Fig. 2:**
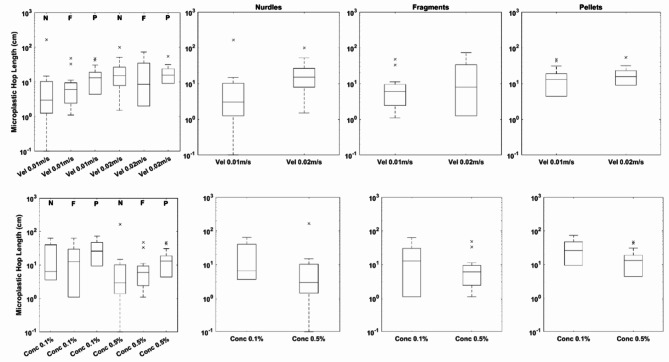
Distribution of microplastic transport rates for the three types of microplastic used within the experiments as a function of flow velocity and bulk microplastic concentration. The left-hand sub plots show all data for comparison with the three sub plots on the right grouped individually for each plastic type for velocity (top) and concentration (bottom).On all graphs the central mark indicates the median, and the bottom and top edges of the box indicate the 25th and 75th percentiles, respectively. The whiskers extend to the most extreme data points not considered outliers, and the outliers are plotted individually using the ‘*x*’ marker symbol.

Although not statistically significant, median microplastic hop length shows a more consistent relationship with both changes to flow velocity and bulk microplastic concentration (Fig. 2). Specifically, there is a positive correlation with flow velocity and median microplastic hop length but a negative correlation with bulk microplastic concentration and median microplastic hop length. These relationships are irrespective of plastic type.

However, given the stochastic nature of particle transport, including microplastics^23,24^, it is better to consider the characteristics of the distribution of parameters rather than median values. Figures 1 and 2 indicate distributions are skewed; there are consistent changes to the skewness of the distribution of microplastic transport rates. There is a negative correlation between the skewness of transport values and both flow velocity (10%, 46% and 64% decrease for nurdles, fragments and pellets respectively) and bulk microplastic concentration (29%, 19% and 65% decrease for nurdles, fragments and pellets respectively) suggesting a decrease in the largest transport rates irrespective of microplastic type. Conversely, for hop lengths there is an inverse relationship with flow velocity and skewness (47%, 59% and 31% decrease for nurdles, fragments and pellets respectively) but a positive relationship between hop length and bulk microplastic concentration (113%, 125% and 113% increase for nurdles, fragments and pellets respectively).

**Fig. 3:**
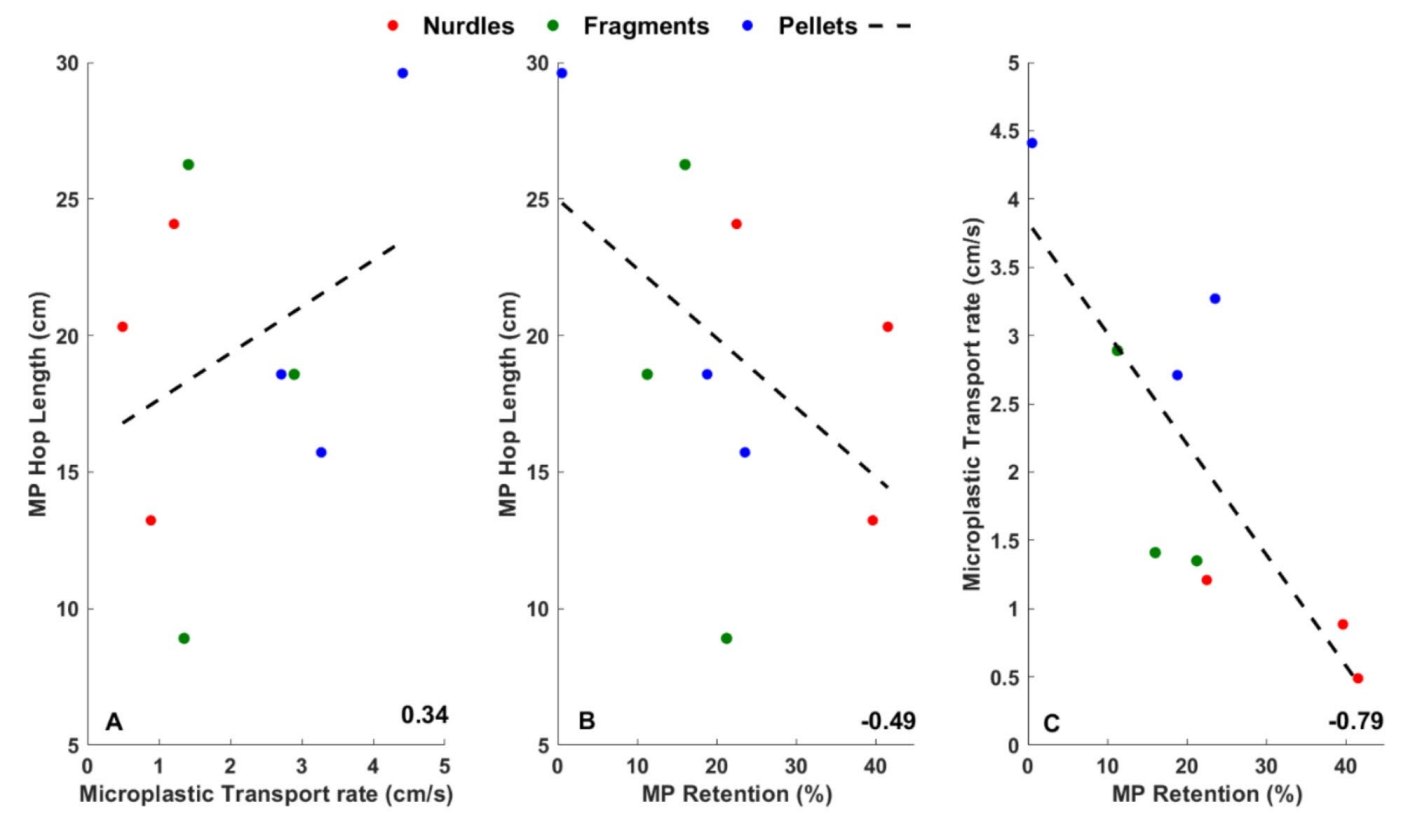
Relationship between average microplastic transport rate (Panel A), hop length (Panel B) and retention within the bed (Panel C). The standard least squares regression is shown by the black dashed line and the Pearson correlation coefficient values are given in the bottom right of the plots.

Once microplastics are entrained there is likely to be a relationship between their transport rate and hop length constrained by the relationship between topographic change and sediment transport at the granular scale^25^. In experiments reported herein, irrespective of whether flow velocity or bulk microplastic concentration are changed there is a positive correlation between microplastic transport rate and microplastic hop length (Fig. 3A). This suggests that plastic particles not only move faster but also further once they are entrained. However, the mobility of microplastics, as evidenced by both their transport rate and hop length is dependent on their type whereby nurdles are least mobile and pellets are most mobile. Further, there is an inverse relationship between microplastic mobility in terms of both transport rate and hop length and their propensity to be retained within the sediment bed where nurdles are retained for longer periods as compared to fragments and pellets (Fig. 3B,C).

### Relationship between bedform and plastic dynamics

**Fig. 4:**
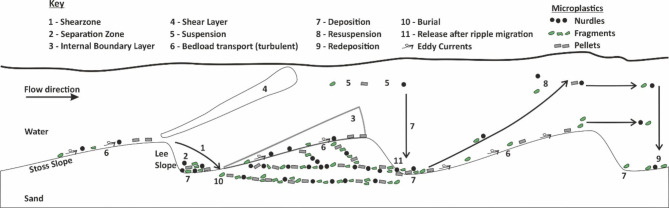
Schematic diagram displaying the different microplastic transport processes as seen in the flume experiments, modified from: Best^26^; Kooi et al^27^; Zhan^28^; Koutnik et al.^29^ Shamskhany et al.^30^. Here points 1–4 link to interactions between bedform morphology flow dynamics, where points 1–2 are also the inter-ripple areas where microplastics tend to gather (Fig. 5a, pale pink ovals). Points 7, 10 and 11 indicate the bedform migration and trapping of microplastics within the sand bed.

Whether or not microplastics will be incorporated deeper into the sediment bed and hence into the longer-term sedimentary structures will depend on the relationship between the bedform and plastic dynamics (Fig. 4).

**Fig. 5:**
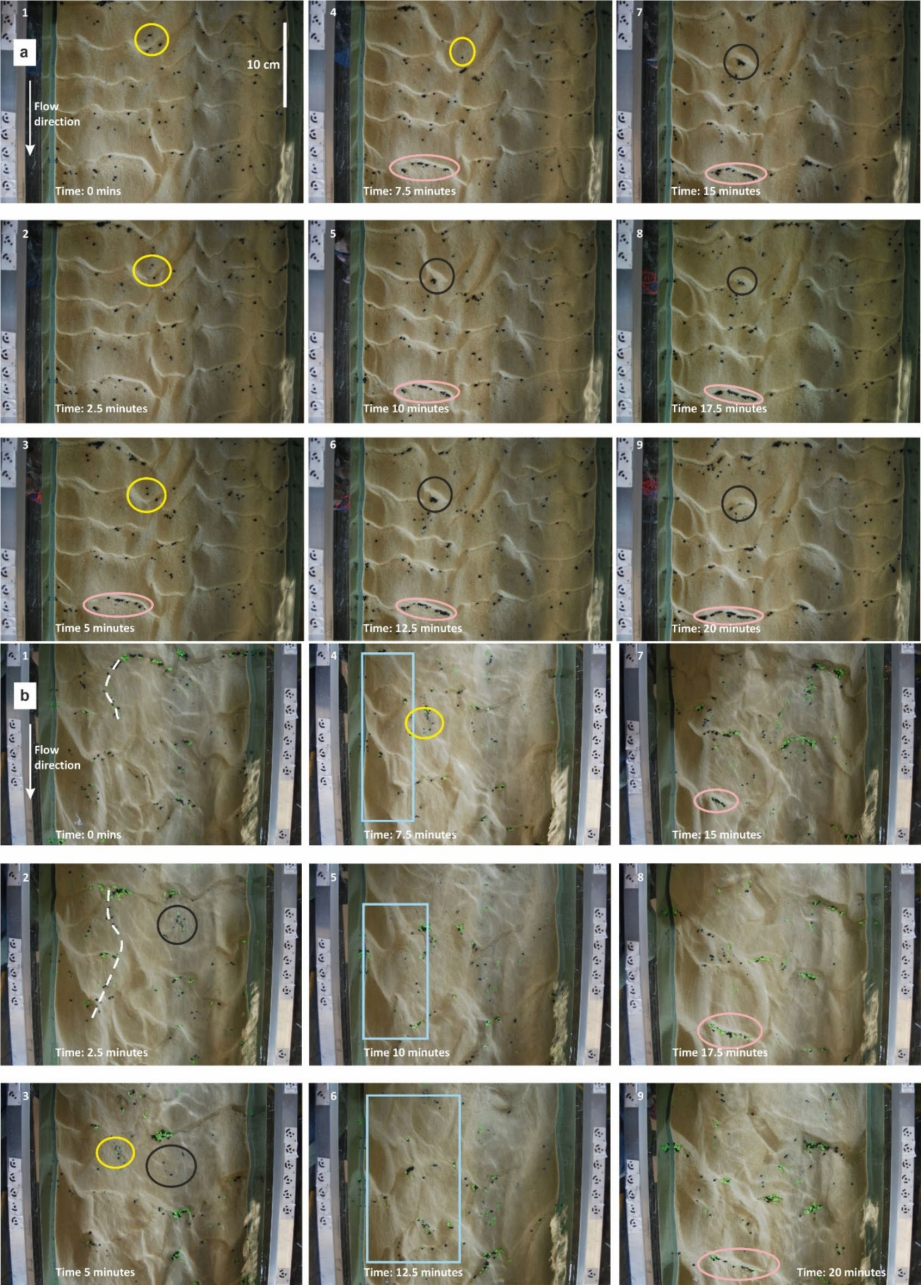
(**a**) Overhead photos taken during the N2 Run which had 0.5% microplastic concentration at a 0.01 m^3^/s flow rate. (**b**). Overhead photos taken during the F3 Run which had 0.5% microplastic concentration at a 0.02 m^3^/s flow rate. Both: Photo 1 was taken at 0 min and photo 9 represents 20 min with the photos in between taken 2.5 min apart. Yellow circles display microplastics being released due to ripple migration; black circles show ripple migration traveling over the microplastics, pink ovals in display microplastics gathering in the inter-ripple areas as the ripples migrate forwards, white lines represent grooves in the sediment due to the preferential travel here by the microplastics, and blue boxes indicate primary current lineation.

Two main mechanisms of microplastic incorporation were observed. The most common process was microplastic burial due to the migration of bedforms over the plastics on or near the surface (Figs. 4 and 5). The second mechanism acted over a shorter timescale whereby the migration of bedforms over the microplastics trapped them in place for the duration of the bedform migration only. As the bedform migrated fully over the microplastics they were released either as single particles or very small groups (Fig. 4). They then either travelled up the stoss slope of the bedform, over the ripple crest and deposited in the inter-ripple areas or they travelled up the stoss slope, into the shear layer and then into the upper flow where they travel in suspension downstream^26^ (Fig. 4). In experiments with higher flow the bedforms had longer stoss slopes and wavelengths allowing for more microplastics to gather in the inter-ripple areas (Fig. 5b), thereby trapping more microplastic in the active layer as these larger bedforms passed over them^22^. Primary current lineation is noted in the faster runs and microplastics dominantly travel along this lineation (Fig. 5b).

**Fig. 6:**
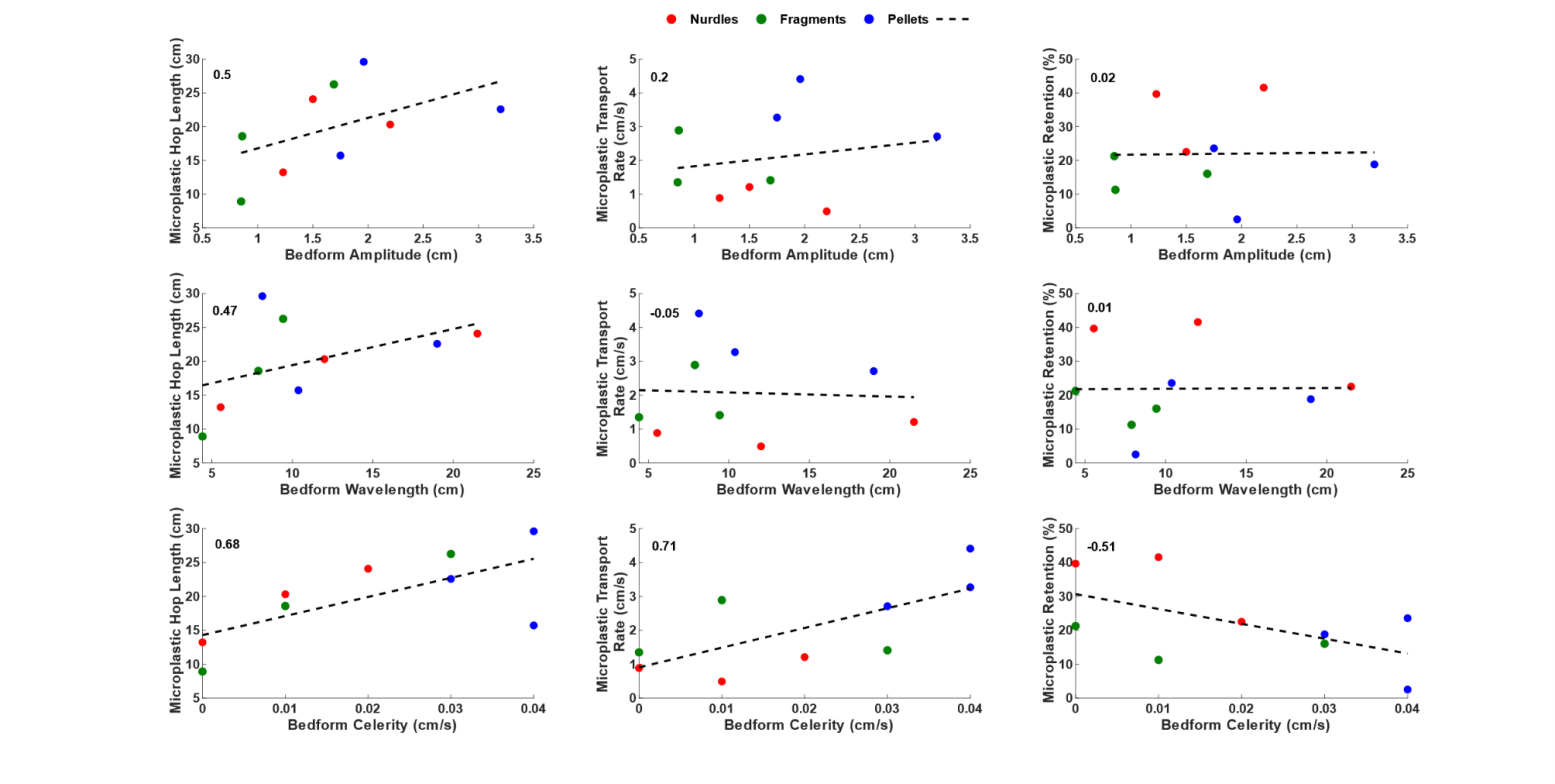
Relationship between average bedform (amplitude, wavelength and celerity) and microplastic parameters (transport rate, hop length and retention within the bed). The standard least squares regression is shown by the black dashed line and the Pearson correlation coefficient values are given in the top left of the plots. Each data point on the subplots represents one flume run.

Figure 6 plots the relationship between average bedform (amplitude, wavelength and celerity) and microplastic parameters (transport rate, hop length and retention within the bed) with Pearson correlation coefficient values shown. Positive correlations are noted between microplastic hop length and all three bedform parameters with the strongest correlation observed between hop length and bedform celerity. The relationship between microplastic transport rate and bedform parameters is more complicated with a weak correlation to bedform amplitude, a very weak correlation with wavelength and a strong positive correlation with bedform celerity. Microplastic retention within the sediment bed shows no correlation with either bedform amplitude or wavelength but a strong negative correlation with bedform celerity. This strongly indicates that it is the speed of the migrating bedform is migrating that controls the flux of microplastics from within sand beds. Further, the type of plastic also appears to be important whereby pellets are not retained within the sediment bed and when entrained within the flow move furthest and fastest. Conversely, nurdles are retained within the bed at higher concentrations but when they are entrained, they are transported comparatively slowly and for shorter distances. Finally, fragments appear to control the size of the bedforms as the amplitude and wavelengths are smaller when compared to the sizes of the bedforms with other microplastic types.

## Discussion

Both field and experimental data have shown that sediment beds can transition from a resilient sink to a source of microplastics dependent on the flow regime, the sediment bed grain size and the microplastic characteristics^8,11,12^. If the river is acting as a sink for prolonged periods of time, the chances of microplastics being preserved within the longer-term sedimentary record increases and hence overall microplastic contamination decreases. This is especially true in sand bed rivers where bedforms can be well preserved in the rock record^20,21^. In experiments reported herein, microplastic retention within the sediment bed has been shown to vary from between 2 and 40%. However, unlike in gravel bed rivers the processes of entrainment and retention of plastics have been shown to vary in response to both microplastic properties as well as bedform dynamics rather than explicitly the flow dynamics.

In relation to microplastic properties, the size and density of the plastics were similar, but shape was changed with the impact of shape having been clearly shown to be important; pellets are seen to be highly mobile as evidenced by both higher transport rates and lower bed retention levels as compared to fragments. In granular sediment beds, enhanced fluid drag on aspherical grains counteracts the enhanced granular friction, partially obscuring the relation between grain shape and bed load transport parameters as both competing effects generally get stronger as grain shape deviates from spherical ^31,32^. In experiments comparing grain shape of natural sediment particles, Cassel et al.,^33^ noted a similar trend as results presented herein whereby compact blade shaped particles, akin to pellets used here, were more mobile in terms of both distance travelled and mean hop velocity, as compared to disc shaped particles, akin to fragments reported herein. Waldschläger and Schüttrumpf^7^ directly assessed the impact of microplastic shape, and they observed a lower statistical significance of the dependence between critical shear stress and shape as compared to the influence of particle density and size. Despite this, they did observe that spherical particles moved before particles of a different shape but with smaller diameters and lower particle densities. Hoellein et al.,^34^ and Akdogan and Guven^35^ reported similar findings where they observed that smooth sided, uniform shaped pellets, like those used within this research, travelled the farthest as compared to sharp sided fragments with more complex and variable shapes. In all studies results were attributed to pellets as having a smaller surface area in contact with the underlying sediment bed, such that there was a lower shear resistance and therefore lower drag force required for particle movement, illustrating that shape of a grain is influential on the transport processes of that grain^36^. It is likely that differential processes responsible for controlling microplastic flux will therefore affect the concentrations observed within sand bed systems whereby pellets will likely have a shorter retention time and lower chance of burial compared to fragments. Given pellets are considered primary microplastics and fragments are usually secondary microplastics there is a need to consider how to best manage their contamination in fluvial sand bed systems, especially legacy plastics which may be mobilised to differing degrees.

Alongside shape effects, relative size between particles (D_i_/D_50_), in this case between the microplastic and sand particles, have also been shown to be important for both entrainment and retention of particles within sediment beds^37–40^. Here, relative size values ranged from 7.94 to 10.08 where ratios greater than 1 indicate microplastic particles that are larger than the sediment bed and hence more exposed to the flow^40^. Where there are high D_i_/D_50_ values essentially the bed is composed of a bimodal distribution with a coarse (microplastic) and fine (sand) fraction. The influence of relative size effects has been represented empirically by ‘hiding functions’ ^41,42^ with an exponent used to quantify the extent to which the relative size effects act to reduce the intrinsic differences in mobility between coarse and fine fractions. Bimodality causes a change in hiding exponent function such as to render all grains equally mobile^39,43^. Consequently, given there are observed differences in microplastic transport rate despite similar sized microplastic grains being used suggests that relative size effects cannot explain microplastic mobility alone. This is supported by Waldschläger and Schüttrumpf^7^, who used a similar size sediment bed with similar D_i_/D_50_ values and observed that large D_i_/D_50_ values corresponded to the greatest deviation from the Shields curve. In these experiments shape has been seen to have a significant effect on microplastic mobility, so we propose that in sand bed rivers the impact of shape is more important in controlling flux than relative size.

Finally, our data have shown that the incorporation and release of microplastic grains within the sediment bed is also controlled by the bedform dynamics. Two different mechanisms formed two different depositional units within the bedform recognised as bottomsets and foresets^44^. The most common process was microplastic burial due to the migration of bedforms over the plastics on or near the surface (Fig. 4). In this instance the plastic particles are likely to be incorporated in lenses tangential to the flow direction forming a bottomset. The second mechanism acted over a shorter timescale whereby the migration of bedforms over the microplastics trapped them in place for the duration of the bedform migration only. In this case microplastics were transported up the stoss slope and deposited on the lee slope forming distinct forset depositional units akin to the processes reported by Russell et al.,^22^ (Fig. 4). Forset deposits are common in sand grained systems where there are a mixture of grain sizes, as in the research here and the sediment results in an upward fining deposit^45–48^. This deposit forms the source of sediment for entrainment during the next discharge wave, but sediment or microplastic entrainment will depend on the depth in which it is buried^47^.

The depth of disturbance and hence potential release of plastics from within the bed is controlled by active layer dynamics^12^. In gravel bed rivers, the active layer is easily defined and typically scaled to a characteristic surface grain size^48–50^ that has also been shown to be appropriate for microplastics^12^. However, in sand bed rivers there is a debate around what constitutes the active layer. Some authors relate it simply to a layer of mobile sediment that moves over the top of the dune to avalanche down the distal face as described by bedform amplitude, whereas some describe it as being the entire bedform, as defined by the bedform celerity^13^. In results reported herein there are weak correlations between bedform amplitude and wavelength and the microplastic flux parameters of transport rate, hop length and retention within the sediment bed. Conversely, there are strong correlations between all three microplastic flux parameters and bedform celerity. This supports the use of bedform celerity as being the most appropriate parameter relating active layer dynamics and their control on microplastic flux in sand bed rivers. The relationship is particularly strong between bedform celerity and microplastic retention in the sediment bed such that there is potential to use the inverse relationship with bedform celerity to predict the likely retention of microplastics within sand bed rivers beyond the conditions presented herein. Microplastics that are deposited as bottomsets are more likely to be retained within the sediment bed for longer as compared to those that form forsets; these are more likely to be eroded from the sediment bed over shorter timescales in response to the passage of multiple bedforms. The latter will depend on bedform celerity which we have shown to be a key control and predictor on microplastic flux in sand bed rivers.

Assuming sand bed rivers can store up to 40% of their microplastic load, indicates the potential for them to be resilient sinks and the retention effectively serves to reduce microplastic transfer from sand bed river systems. Data reported herein is suggestive that it is the bedform dynamics that control flux of microplastics rather than being directly controlled by flow characteristics as is the case with gravel bed rivers. However, whilst these relationships hold true for steady flow conditions, well described bedform phase diagrams fall short in predicting bedform dynamics under time varying flow such as those experience during a flood event^51,52^. As such further research is needed to understand how the interplay between bedform dynamics and flow characteristics and their controls on microplastic flux will evolve under time varying flow. Further, it is recognised that contamination in real rivers is often far more complex and characterised by microplastics which have a range of sizes, densities and morphologies. Given these characteristics will affect microplastic propensity to be eroded from, or retained within, the sediment bed as well as their entrainment characteristics once they are eroded, future research should be directed towards understanding the implications of this for modelling their fate.

## Materials and methods

Flume experiments were conducted to quantify the relationship between bedform dynamics and microplastic incorporation and retention within sand bed rivers (Table 1). Experiments were performed within a glass sided, flow recirculating flume of rectangular cross section (8 m × 0.5 m × 0.5 m). To prevent scour and to induce turbulent boundary conditions, 2 m of immobile sediment was placed directly downstream of the water inlet and no measurements were made in the final 1.5 m of the flume to avoid draw-down effects. This gave an effective working length of 5.5 m, which was covered with 0.4 mm sand to depth of 0.16 m.

**Table 1:** Experimental conditions.

Run	Microplastic type	Total microplastic input	Flow velocity	Corey shape factor	D_i_/D_50_
N1	Nurdles	630 g	0.01 m^3^/s	1.06	9.85
N2		2.1 k g	0.01 m^3^/s		
N3		2.1 k g	0.02 m^3^/s		
F1	Fragments	630 g	0.01 m^3^/s	0.36	10.08
F2		2.1 kg	0.01 m^3^/s		
F3		2.1 kg	0.02 m^3^/s		
P1	Pellets	630 g	0.01 m^3^/s	0.50	7.94
P2		2.1 kg	0.01 m^3^/s		
P3		2.1 kg	0.02 m^3^/s		

The Corey Shape factor is calculated as $CSF= \frac{c}{\sqrt{ab}}$ where a, b and c represent the long, intermediate and short axis of each measured plastic grain respectively. For the relative size ratio (*D*_*i*_*/D*_*50*_) *D*_*i*_ is determined using the nominal diameter by Wadell^53^ which determines the equivalent sphere diameter of a particle based on its three side lengths a, b, and c: $Di=\sqrt[3]{} acb$*. D*_*50*_ is the median size of the sediment bed. Reported values for the Corey Shape Factor and *D*_*i*_*/D*_*50*_ are the average values calculated from measurements of 20 individual microplastic grains for each microplastic type.

Three types of microplastic were used within the experiments with plastic type and concentration chosen to represent those reportedly found within fluvial environments^12,22^; (i) recycled rigid polyvinyl chloride (PVC) microplastic nurdles ranging from 3 to 5 mm (D_50_ (median size) of 3.8 mm with a density of 1.33 g/cm^3^)); (ii) rigid polycarbonate fragments ranging from 0.5 to 5 mm (D_50_ of 4 mm with a density of 1.2 g/cm^3^)) and (iii) rigid polycarbonate pellets ranging from 3 to 4 mm (D_50_ of 3 mm with a density of 1.23 g/cm^3^). The plastics were not treated in any way prior to being added into the flume where they were incorporated into the sediment mixture at either a 0.1 or 0.5% concentration by mass following concentrations used by Russell et al.,^22^. Therefore, this work has not used fibres as they have a significantly lower density. By excluding the influence of density from this work, we can determine if the MP shape influences the migration of bedforms.

### Experimental procedure

During each experiment the flume was set to a zero slope and the bed screeded to match the flume slope. Two experimental phases were then run; an initial 45-min steady flow period of 0.01 m^3^/s^-1^ was run until equilibrium bedform conditions were attained which was followed by another 45-min experimental observation period. In this phase flow rate was set to either 0.01 m^3^/s^-1^ (experiments N1-N2, F1-F2 and P1-P2) or 0.02 m^3^/s^-1^ (experiments N3, F3 and P3) and in all cases, flow was uniform through the test section, with no measured acceleration or deceleration along the length of the flume and a relatively stable water surface. The first run with each of the microplastic types were run at 0.1% concentration of microplastics where they were fed at the upstream end of the working section at the end of the equilibrium flow period. During these runs the plastics became incorporated into the sediment bed and were not removed for subsequent runs. Consequently, for the second and third runs or each plastic type microplastics were mixed throughout the whole depth of the sediment bed where additional plastics were added to make a concentration of 0.5% by mass.

Bed morphology was captured using video and photography throughout each run. Videos were taken looking into the flume from both sides and top-down^54,55^. Time lapse photography was taken every 2.5 min using a Digital Single Lens Reflex (DSLR) camera looking top-down into the flume with two LED spotlights facing the flume on either side providing illumination of the flume bed^55^. The side-on imagery was used to manually measure the amplitude of the bedforms^54^ whilst the top-down imagery was used to measure the migration rates of the bedforms and the MPs collecting 500 readings. Sediment traps at the downstream end of the working section was emptied at the end of each run and total sediment and microplastic flux rates were calculated. Microplastic retention in the sediment bed was calculated as the mass collected in the sediment trap at the end of each experiment as a % of the original microplastic input.

A one-way Kruskal–Wallis ANOVA test was run between (i) individual plastic types (N1,N2,N3; F1,F2,F3; P1,P2,P3); and (ii) all plastic types where experimental conditions were the same (experiments to test for the impact of changing flow velocity (N2,N3,F2,F3,P2,P3) experiments to test for the impact of changing bulk microplastic concentration (N1,N2,F1,F2,P1,P2)) to test for statistical significance in any observed differences.

## Data Availability

All datasets are available from the corresponding authors upon reasonable request.
